# 2017 ASCO及WCLC晚期非鳞NSCLC抗血管生成治疗进展

**DOI:** 10.3779/j.issn.1009-3419.2018.05.12

**Published:** 2018-05-20

**Authors:** 莲芳 倪, 立功 聂

**Affiliations:** 1 100034 北京，北京大学第一医院老年内科 Department of Geriatric Medicine, Peking University First Hospital, 100034 Beijing, China; 2 100034 北京，北京大学第一医院呼吸和危重症科 Department of Respiratory and Critical Care Medicine, Peking University First Hospital, 100034 Beijing, China

**Keywords:** 肺肿瘤, 抗血管生成治疗, 表皮生长因子受体酪氨酸激酶抑制剂, Lung neoplasms, Antiangiogenic therapy, Epidermal growth factor receptor tyrosine kinase inhibitor

肺癌是全球威胁人类生命健康最大的恶性肿瘤，在我国也是如此，其发病率和病死率占所有恶性肿瘤的18.74%和25.24%，并有逐年上升的趋势^[1]^。但是肺癌预后并不尽如人意，5年生存率仅17%^[2]^。近年来出现的小分子靶向药物极大地改善了驱动基因阳性晚期非小细胞肺癌（non-small cell lung cancer, NSCLC）患者的预后，但驱动基因阴性患者的治疗手段仍非常有限。血管生成在恶性肿瘤的生长、发展和转移过程中起关键作用，抗血管生成药物作用于肿瘤微环境，通过抑制肿瘤血管的生成，抑制肿瘤生长和转移。在化疗的基础上联合抗血管生成药物突破了肿瘤治疗的瓶颈，提高了化疗的疗效和患者的生存获益，已成为晚期NSCLC的标准治疗选择之一。2017年美国临床肿瘤学会（American Society of Clinical Oncology, ASCO）及世界肺癌大会（World Conference on Lung Cancer, WCLC）会议上发布的抗血管生成领域的临床研究带给我们一些新的认知，现介绍如下。

## 1 贝伐珠单抗跨线治疗能否带来更多获益？

ASCO上公布的AvaALL研究^[3]^对贝伐珠单抗的跨线治疗进行了探索。这是一项开放标签、随机Ⅲ期研究，探讨晚期非鳞癌NSCLC一线贝伐珠单抗（Bev）和化疗（Chemo）后疾病进展（progressive disease, PD）的患者跨线标准治疗（standard of care, SOC）加或不加贝伐珠单抗治疗的疗效和安全性。研究共入组晚期非鳞癌NSCLC符合意向治疗（intention-to-treat, ITT）的患者485例，排除所有已知表皮生长因子受体（epidermal growth factor receptor, EGFR）突变阳性患者，中位随访9.4个月。主要终点为总生存期（overall survival, OS）（PD1-死亡），次要终点为无进展生存期（progression free survival, PFS）（PFS2: PD1-PD2; PFS3: PD2-PD3）、疾病进展时间（time to progress, TTP）（TTP2: PD1-PD2; TTP3: PD2-PD3）及安全性。二线标准治疗类型包括厄洛替尼*vs*多西他赛*vs*培美曲塞。结果显示，贝伐珠单抗跨线治疗仅仅改善了PFS3、TTP2和TTP3，主要终点OS未达到统计学意义[中位OS 11.9个月（Bev+SOC）*vs* 10.2个月（SOC）；*P*=0.104, 4]；同时也未发现新的安全信号。基于上述结果，笔者认为贝伐珠单抗跨线治疗尚需进一步临床验证，目前贝伐珠单抗最佳治疗模式仍是BEYOND模式（一线诱导+维持至PD或毒性不可耐受）。

## 2 抗血管生成药物在晚期NSCLC三线治疗的探索

晚期NSCLC的三线治疗手段较为有限，且疗效不足和无统一标准。安罗替尼是我国自主研发的一种口服的新型小分子多靶点酪氨酸激酶抑制剂（tyrosine kinase inhibitors, TKIs），具有抗肿瘤血管生成和抑制肿瘤生长的作用。ASCO会议公布的韩宝惠教授团队的ALTER 0303研究^[4]^对其在晚期NSCLC三线治疗进行了探索。这是一项全国多中心、随机、双盲、安慰剂对照Ⅲ期研究，旨在评价安罗替尼作为晚期NSCLC三线治疗的疗效和安全性。共纳入437例既往至少接受过两次全身化疗的晚期NSCLC患者，随机接受安罗替尼（*n*=294）或安慰剂（*n*=143）治疗，直至疾病进展或不可耐受的毒性。主要研究终点为OS。次要终点包括PFS、客观缓解率（objective response rate, ORR）、疾病控制率（disease control rate, DCR）以及安全性。结果显示，安罗替尼组的OS显著延长（9.6 mo *vs* 6.3 mo, *P*=0.001, 8），PFS（5.4 mo *vs* 1.4 mo, *P* < 0.000, 1）、ORR（9.2% *vs* 0.7%, *P* < 0.000, 1）和DCR也显著优于对照组（81.0% *vs* 37.1%, *P* < 0.000, 1）；不良事件发生率与对照组相似，显示了良好的安全性。该研究表明，安罗替尼作为晚期NSCLC的三线治疗，能够带来PFS和OS双重获益，可作为重要的三线治疗选择。

## 3 抗血管生成联合表皮生长因子受体酪氨酸激酶抑制剂（epidermal growth factor receptor-tyrosine kinase inhibitors, EGFR-TKIs）的新进展

临床前研究显示阻断VEGFR和EGFR有协同作用，抗血管生成药物与EGFR-TKIs联合治疗有敏感突变的NSCLC之前有过各种探索，比如日本的Ⅱ期临床研究^[5]^，贝伐珠单抗联合厄洛替尼治疗未化疗过的携带驱动基因突变的NSCLC，联合治疗组PFS获益明显（16.0 mo *vs* 9.7 mo, HR=0.54, *P*=0.001, 5），还有欧洲胸部肿瘤平台的开放性的单臂Ⅱ期BELIEFL研究^[6]^。新近的FLAURA研究^[7]^显示，第三代EGFR-TKI奥西替尼一线治疗*EGFR*突变阳性的局部晚期或转移性NSCLC，与第一代EGFR-TKI相比（吉非替尼或厄洛替尼），可进一步延长患者的中位无进展生存（mPFS）（18.9 mo *vs* 10.2 mo），降低疾病进展和死亡风险达54%（HR=0.46; 95%CI: 0.37-0.57; *P* < 0.000, 1），给晚期肺癌患者带来巨大的生存获益。那么，抗血管生成联合第三代EGFR-TKI能否有更大的获益？ASCO汇报了奥希替尼联合贝伐珠单抗初始治疗EGFR突变型肺癌患者的Ⅰ期临床研究^[8]^，这项单中心Ⅰ期剂量递减研究显示，在17例可评估的患者（≥2次影像学评价）中，76%（13/17）的患者获得部分或完全缓解。同时，奥希替尼+贝伐珠单抗初步安全性良好，3级治疗期间出现的不良事件（treatment-emergent adverse events, TEAEs）主要是高血压，发生率19%（4/21）。这一研究的最终结果值得我们期待。

Fuquintinib是国内自主研发的口服小分子抗血管生成药物，具有高度选择性，可长效抑制VEGFR。2017国际肺癌研究协会（International Association for the Study of Lung Cancer, IASLC）世界肺癌大会（World Conference on Lung Cancer, WCLC）汇报了我国陆舜教授等^[9]^“Fruquintinib联合吉非替尼治疗Ⅲb期/Ⅳ期伴有*EGFR*敏感突变NSCLC的Ⅱ期研究”。这是一项单臂开放多中心研究，共入组26例初治Ⅲb期/Ⅳ期伴有*EGFR*敏感突变NSCLC患者。主要观察安全性、耐受性和ORR。在可评价疗效的17例患者中，13例部分缓解（partial response, PR），4例疾病稳定（stable disease, SD），ORR为76.5%，DCR为100%。3级及以上TEAEs发生率为30.8%（8/26），主要为肝酶ALT升高。鉴于该试验结果的临床疗效和安全性，该联合用药值得进一步研究。

## 4 贝伐珠单抗治疗肺癌脑转移的现实世界结果

脑转移是晚期NSCLC常见的转移部位，也是导致患者死亡的主要原因之一。由于抗血管生成药物的出血风险，很多临床试验把脑转移作为入组的排除标准。但近年来很多的荟萃分析及临床研究发现，贝伐珠单抗的治疗并不增加脑转移患者中枢神经系统的出血几率。2017 IASLC WCLC汇报的法国Bennouna等的“贝伐珠单抗联合化疗治疗晚期NSCLC和脑转移”的研究^[10]^进一步验证了贝伐珠单抗对于肺癌脑转移患者的安全性。这是一项非干预前瞻性全国多中心的研究，描述在常规临床实践中晚期NSCLC患者接受一线化疗+贝伐珠单抗的情况，主要终点为用药情况、PFS、OS和安全性。共入组有效分析患者407例，其中伴脑转移患者84例，占20.6%。99.5%的患者联用含铂双药化疗。中位随访10.8个月。结果显示，伴或不伴脑转移的两组患者在mPFS、mOS及不良反应方面无统计学差异。即就临床受益（生存和安全性）而言，是否伴有脑转移的NSCLC患者接受一线化疗+贝伐珠并无差异，脑转移不是独立的预后因素，也不是OS或PFS的预测因素，贝伐珠单抗的副作用的严重程度与已知的一致。

## 5 现实世界贝伐珠单抗如何应用最大获益？

贝伐珠单抗治疗一线治疗晚期非鳞NSCLC的获益已经多个临床试验证实，那么，现实世界的应用获益如何呢？2017 IASLC WCLC发布了我国李峻岭教授团队^[11]^的现实世界研究。研究收集了149例晚期非鳞NSCLC患者数据进行回顾性分析，其中一线应用研究贝伐珠单抗治疗者62例，占41.6%。主要终点为PFS，次要终点为ORR、DCR和安全性。中位随访10.7个月。结果显示，在总人群及*EGFR*或*ALK*驱动基因阴性亚组，PFS均显著获益（9.7 mo *vs* 7.0 mo, HR=0.52, *P*=0.018, 4; 11.3 mo *vs* 5.5 mo, HR=0.43, *P*=0.023, 4），ORR、DCR均有改善趋势。两组均未发现新的安全事件。现实世界的研究证实一线含贝伐珠单抗方案治疗NS-NSCLC优于不含贝伐珠单抗的方案。

含贝伐珠单抗方案一线或后续治疗有较好的疗效和耐受性，但缺乏直接证据证明一线应用（1L）优于后续应用（LL）。2017 IASLC WCLC我国李峻岭教授团队的研究^[12]^回答了这一问题。研究回顾性分析159例NSCLC晚期NSCLC一线或后续应用含贝伐珠单抗化疗方案的治疗效果。主要终点为PFS，次要终点为ORR、DCR和安全性。1L与LL两组间患者特征基本平衡，中位随访10.7个月。与LL组比较，1L组的中位PFS明显延长（9.7 mo *vs* 4.1 mo, HR=0.28, 95%CI: 0.15-0.52, *P* < 0.000, 1）。ORR和DCR有改善趋势。亚组分析显示，与LL组比较，1L组的EGFR/ALK野生型（WT）亚组（mPFS: 11.3 mo *vs* 3.4 mo, HR=0.2, 95%CI: 0.08-0.48, *P* < 0.000, 1）和WT+突变未知亚组（mPFS: 11.3 mo *vs* 3.4 mo, HR=0.25, 95%CI: 0.12-0.51, *P* < 0.000, 1）获益更明显，ORR和DCR结果也是一致的。没有发现不可预知的安全性问题。该研究表明，一线应用贝伐珠单抗较后续应用有更为优越的疗效，特别是对EGFR/ALK野生型或未知的NSCLC。

综上，目前贝伐珠单抗应用的最佳模式是一线+维持，跨线应用有待进一步的研究。安罗替尼作为晚期NSCLC的三线治疗，能够带来PFS和OS双重获益，可作为重要的三线治疗选择。同时阻断VEGFR和EGFR可以延长PFS，是延迟EGFR-TKIs耐药的一种选择。有脑转移的非鳞NSCLC患者，应用贝伐珠单抗同样安全、有效。一线应用贝伐珠单抗较单纯化疗更好；一线应用贝伐珠单抗较后续应用更好。
